# Ferroptosis as a Major Factor and Therapeutic Target for Neuroinflammation in Parkinson’s Disease

**DOI:** 10.3390/biomedicines9111679

**Published:** 2021-11-12

**Authors:** Chih-Jan Ko, Shih-Ling Gao, Tsu-Kung Lin, Pei-Yi Chu, Hung-Yu Lin

**Affiliations:** 1 Department of General Surgery, Changhua Christian Hospital, 135 Nanhsiao Street, Changhua 500, Taiwan; cch91681@gmail.com; 2 School of Medicine, Kaohsiung Medical University, Kaohsiung 807, Taiwan; 3 Department of Nursing, Changhua Christian Hospital, 135 Nanhsiao Street, Changhua 500, Taiwan; 109300@cch.org.tw; 4 Center for Mitochondrial Research and Medicine, Kaohsiung Chang Gung Memorial Hospital, Chang Gung University College of Medicine, Kaohsiung 833, Taiwan; tklin@cgmh.org.tw; 5 Center of Parkinson’s Disease, Kaohsiung Chang Gung Memorial Hospital, Chang Gung University College of Medicine, Kaohsiung 833, Taiwan; 6 College of Medicine, National Chung Hsing University, Taichung 402, Taiwan; 7 Department of Pathology, Show Chwan Memorial Hospital, Changhua 500, Taiwan; 8 School of Medicine, College of Medicine, Fu Jen Catholic University, New Taipei City 242, Taiwan; 9 Department of Health Food, Chung Chou University of Science and Technology, Changhua 510, Taiwan; 10 National Institute of Cancer Research, National Health Research Institutes, Tainan 704, Taiwan; 11 Research Assistant Center, Show Chwan Memorial Hospital, Changhua 500, Taiwan

**Keywords:** Parkinson’s disease, ferroptosis, neuroinflammation, iron, lipid peroxidation, antioxidant defense

## Abstract

Mounting evidence suggests that ferroptosis is not just a consequence but also a fundamental contributor to the development and progression of Parkinson’s disease (PD). Ferroptosis is characterized as iron-dependent regulated cell death caused by excessive lipid peroxidation, leading to plasma membrane rupture, release of damage-associated molecular patterns, and neuroinflammation. Due to the crucial role of intracellular iron in mediating the production of reactive oxygen species and the formation of lipid peroxides, ferroptosis is intimately controlled by regulators involved in many aspects of iron metabolism, including iron uptake, storage and export, and by pathways constituting the antioxidant systems. Translational and transcriptional regulation of iron homeostasis and redox status provide an integrated network to determine the sensitivity of ferroptosis. We herein review recent advances related to ferroptosis, ranging from fundamental mechanistic discoveries and cutting-edge preclinical animal studies, to clinical trials in PD and the regulation of neuroinflammation via ferroptosis pathways. Elucidating the roles of ferroptosis in the survival of dopaminergic neurons and microglial activity can enhance our understanding of the pathogenesis of PD and provide opportunities for the development of novel prevention and treatment strategies.

## 1. Introduction

Parkinson’s disease (PD) is the second most common neurodegenerative disease affecting populations globally. The global death due to PD increased by approximately 40% between 2005 and 2015 [1]. According to pathological studies, a reduction in the number of dopaminergic neurons and an abnormal accumulation of α-synuclein (α-syn) result in a shortage of dopamine from the substantia nigra–striatum pathway, causing clinical symptoms such as tremor, bradykinesia, rigidity, and postural balance disorders [2,3]. Although the pathology of PD remains unclear, compelling evidence from clinical, preclinical, and epidemiological studies suggests that neuroinflammation and oxidative stress may play central roles in PD pathogenesis [4,5,6]. 

**Figure 1:**
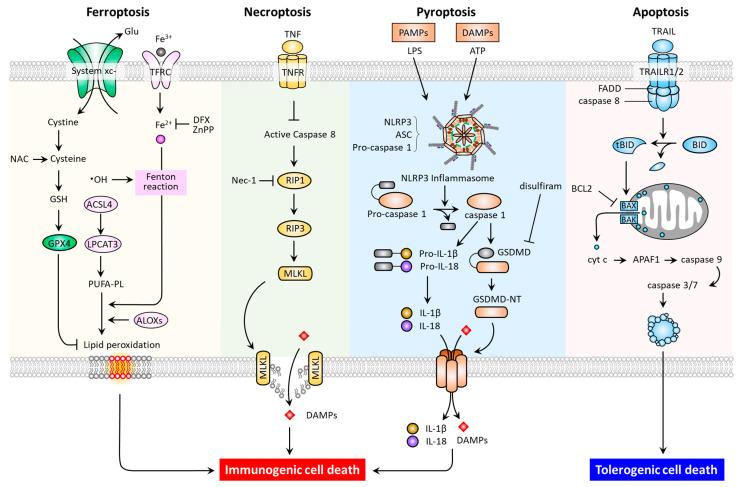
Molecular pathways and immunogenic properties of ferroptosis, necroptosis, pyroptosis, and apoptosis. ACSL4, acyl-CoA synthetase long chain family member 4; ALOXs, arachidonate lipoxygenases; ASC, apoptosis-associated speck-like protein containing a CARD (ASC); LPCAT3, lysophosphatidylcholine acyltransferase 3; TFRC, transferrin receptor; APAF-1, apoptotic peptidase activating factor 1; BAK, Bcl-2 homologous antagonist/killer; BAX, Bcl-2-associated X; BCL2, B-cell lymphoma 2; BID, BH3 interacting domain death agonist; DFX, deferoxamine; tBID, truncated BH3 interacting domain death agonist; FADD, Fas-associated protein with death domain; GSDMD, gasdermin D; MLKL, mixed lineage kinase domain-like pseudokinase; Nec-1, necrostatin-1; NLRP3, Nod-like receptor 3; RIP1, receptor-interacting protein kinase 1; RIP3, receptor-interacting protein kinase 3; TNF, tumor necrosis factor; TNFR, tumor necrosis factor receptor; TRAIL, tumor necrosis factor-related apoptosis-inducing ligand; TRAILR1, tumor necrosis factor-related apoptosis-inducing ligand receptor 1.

In multicellular organisms, regulated cell death (RCD) is a function necessary for the normal development and maintenance of tissue homoeostasis, as well as to eliminate damaged cells. RCD is controlled death that involves tightly structured signaling cascades and molecularly defined effector mechanisms [7]. In addition to apoptosis and necroptosis, studies have recently revealed new types of RCD, including pyroptosis and ferroptosis [8,9,10,11]. These RCD modes have distinct cellular morphological, biochemical (Table 1), and signaling pathway (Figure 1) features. 

As one of the most commonly studied forms of RCD, apoptosis may be triggered by both extrinsic (death receptor-activated) and intrinsic (mitochondrial or B-cell lymphoma 2 (BCL-2) regulated) pathways. The extrinsic pathway can be activated by ligation of the tumor necrosis factor receptor (TNFR) superfamily members, which promote adaptor proteins (e.g., FADD) to activate caspase-8, and then the downstream effector caspase-3 [12]. The intrinsic pathway can be induced by intrinsic stress (growth factor deprivation, DNA damage, and endoplasmic reticulum stress), and BH3-only proteins such as BCL2 binding component 3 (BBC3, also known as PUMA), phorbol-12-myristate-13-acetate-induced protein 1 (PMAIP1, also known as NOXA), BCL2 Like 11 (BCL2L11, also known as BIM), BH3 Interacting Domain Death Agonist (BID), and the BCL2 associated agonist of cell death (BAD) [13,14]. To illustrate, p53-upregulated PUMA can bind with high affinity to BCL-2, thereby liberating BAX/BAK to mitochondria. This leads to the formation of mitochondrial outer membrane permeabilization (MOMP), and the released cytochrome c then binds to apoptotic peptidase activating factor 1 (APAF1) to form apoptosome, and the resultant apoptosis. Under endoplasmic reticulum stress, the conformational activation of BAX/BAK occurs at the mitochondria, thereby relaying the signaling required for the assembly of apoptosome [15]. In terms of necroptosis, the inducers have been identified as tumor necrosis factor (TNF), the CD95 receptor/Fas ligand complex, and other members of the TNF superfamily [7]; while receptor-interacting protein kinase 1 (RIP1), RIP3, and mixed lineage kinase domain-like pseudokinase (MLKL) are proteins required for the activation of necroptosis. In response to death receptor activation, the binding of RIP1 to RIP3 triggers the formation of necrosomes, resulting in MLKL activation [11]. As a necroptotic effector, the activated MLKL translocates to the plasma membrane causing membrane lysis and subsequent cell death. Of note, necrostatin-1 has been reported to suppress necroptosis via inhibition of RIP1 activity [16]. With regards to pyroptosis, the predominant hallmarks are the activation of the inflammasome, a cytosolic multiprotein complex accounting for the release of interleukin-1 beta (IL1B) and IL18, the formation of apoptosis-associated speck-like protein containing a CARD (ASC), and the activation of proinflammatory cascades [17]. In general, when pattern recognition receptors (PRRs, e.g., nod-like receptor 3 (NLRP3) and absent in melanoma like receptor 2 (AIM2)) first identify various danger signals, they proceed to activate pro-caspase-1 cleavage and ASC recruitment in order to assemble inflammasomes. The activated caspase-1 acts to cleave the pyroptosis executor gasdemin D (GSDMD) at site Asp275 to free the N-terminal domain (GSDMD-MT) and generate nonselective pores on the cell membrane. Meanwhile, caspase-1 cleaves and activates the precursors of IL1B and IL18 to generate mature IL-1β and IL-18. The intracellular contents are subsequently released via GSDMD-MT-caused pores, leading to pyroptosis [17]. Additionally, an inflammasome-independent, noncanonical pathway mediated by a caspase-1/4/5/11-cleaved GSDMD-MT has been identified [18,19,20]. Thereafter, the drug disulfiram was approved by the Food and Drug Administration (FDA) as a pyroptosis inhibitor via targeting GSDMD [21]. Indeed, after being recently recognized as a distinct type of RCD, further investigation of ferroptosis offers promise for the discovery of novel treatments for PD.

**Table 1:** Morphological and immune features of ferroptosis, apoptosis, necroptosis, and pyroptosis.

	Morphological Features	Representative Inhibitor	Immune Features	Reference
**Ferroptosis**	Small mitochondria	Iron chelators	ICD	[22,23,24]
	Vanishing mitochondrial crista	GPX4		
	OMM rupture	HO-1 inhibitor		
	Normal nucleus Normal cell size			
**Necroptosis**	Swollen mitochondria Release of cytoplasmic constituents Plasma membrane rupture Chromatin condensation Swollen cell	Nec-1	ICD	[7,11]
**Pyroptosis**	Unaltered mitochondria Pore formation on plasma membrane Inflammasome formation Chromatin condensation Swollen cell	Disulfiram	ICD	[17,18,19,20]
**Apoptosis**	Unaltered mitochondria Apoptotic bodies Cytoskeletal disintegration Chromatin condensation Shrinkage of cell	BCL2	TCD	[13,14,15]

BCL2, B-cell lymphoma 2; GPX4, glutathione peroxidase 4; HO-1, heme oxygenase-1; ICD, immunogenic cell death; Nec-1, necrostatin-1; OMM, outer membrane of mitochondria; TCD, tolerogenic cell death.

Dixon et al. first illustrated the process of ferroptosis [22], which refers to iron-dependent cell death caused by lipid peroxidation and subsequent plasma membrane rupture [24]. Being distinct from other types of RCD, ferroptosis does not present the characteristic cellular swelling observed in necroptosis and pyroptosis, nor the cellular shrinkage and formation of apoptotic bodies exhibited in apoptosis (Table 1). In terms of organellar morphology, ferroptosis does not exhibit chromatin condensation in the nucleus or cytoskeletal disintegration. However, it manifests in distinct disorganization of mitochondria, including mitochondrial shrinkage, the vanishing of mitochondrial crista, and the rupturing of the outer mitochondrial membrane (OMM). A growing body of research has led to the identification of an intricate signaling pathway which controls ferroptosis by preventing iron accumulation and lipid peroxidation, or by disturbing the antioxidant defensive system. Indeed, the brain is the main tissue in which iron accumulates [25]. The brain has the highest levels of polyunsaturated fatty acids (PUFAs), which are recognized as lipid peroxide precursors [26]. Furthermore, there is a close correlation of lipid peroxidation with neurodegeneration [27]. During the disease progression of PD, the dysregulation of iron metabolism is closely associated with cellular damage and oxidative stress, while cellular ferroptosis has notably been observed in dopaminergic neurons in both in vivo and ex vivo PD models [28]. As such, it is significant that ferroptosis-targeting agents have been reported to exert positive effects in several preclinical settings and in patients with PD (Table 2), representing positive steps in the development of novel PD therapeutic modalities. In light of this, we aimed to review the latest research on ferroptosis to further the understanding of its pathogenesis and to propose potential targets for the treatment of PD. 

## 2. Iron Accumulation and Lipid Peroxidation

**Figure 2:**
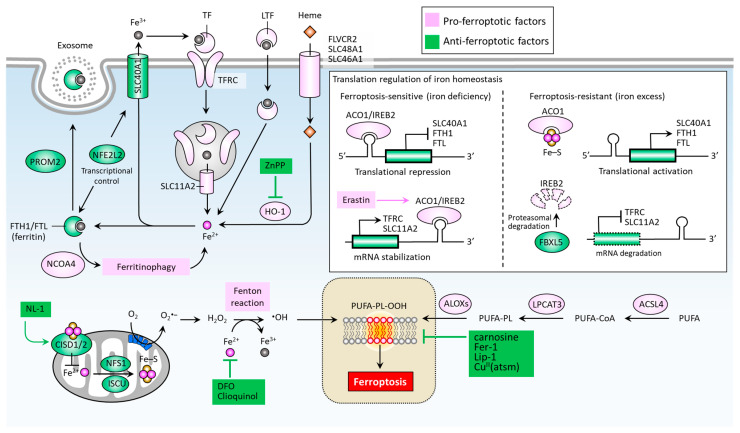
Molecular pathways of iron metabolism and lipid peroxidation in ferroptosis. Ferroptosis is mainly activated by iron accumulation-mediated lipid peroxidation, which is determined by key factors accounting for the import, export, and metabolism of iron and the formation of lipid peroxides (PUFA-PL-OOH). **∙**OH, hydroxyl radical; ACO1, aconitase 1; ACSL, acyl-CoA synthetase long chain family members; ALOXs, lipoxygenases; CISD1/2, CDGSH iron sulfur domain 1/2; Cu^II^ (atsm), diacetyl-bis(4-methyl-3-thiosemicarbazonato) copper^II^; DFO, desferoxamine; FBXL5, F-box and leucine-rich repeat protein 5; FTH1, ferritin heavy chain 1; FTL, ferritin light chain; H_2_O_2_, hydrogen peroxide; HO-1, heme oxygenase-1; IREB2, iron response element binding protein 2; ISCU, iron-sulfur cluster assembly enzyme; LPCAT3, lysophospholipid acyl-transferase 3; LTF, lactotransferrin; NCOA4, nuclear receptor co-activator 4; NFE2L2, nuclear factor erythroid 2 like 2; NFS1, NFS1 cysteine desulfurase; PROM2, prominin 2; PUFA-PL-OOH, lipid peroxides generated from polyunsaturated fatty acid-containing phospholipids; SLC11A2, solute carrier family 11 member 2; SLC40A1, solute carrier family 40 member 1; SCL46A1, solute carrier family 46 member 1; SCL48A1, solute carrier family 48 member 1; TF, transferrin; TFRC, transferrin receptor.

Iron accumulation and lipid peroxidation are two key hallmarks of ferroptosis [22]. Iron is an important trace element, while an aberrant distribution or content of iron within the body may lead to physiological disorders. Iron imported into a cell can be mediated by transferrin (TF) via the transferrin receptor (TFRC) (Figure 2) [49]. Iron-loaded serotransferrin-TFRC complexes are internalized through endosomes, where they release iron (Fe^2+^) into the cytoplasm through solute carrier family 11 member 2 (SLC11A2), leading to increased iron accumulation and the subsequent induction of ferroptosis [49]. Additional sources of iron come from lactotransferrin (LTF) and heme, provided through differing import mechanisms in the cell membrane [24]. Iron export, on the other hand, as mediated by solute carrier family 40 member 1 (SLC40A1), effectively inhibits ferroptosis [50]. The knockdown of TFRC may inhibit erastin-induced ferroptosis [51], while heme oxygenase-1 (HO-1) can act to accelerate erastin-induced ferroptosis by supplementing iron [52]. Ferritin acts as an iron storage protein complex composed of ferritin heavy chain 1 (FTH1) and ferritin light chain (FTL) [53]. Brown et al. reported that prominin2 (PROM2) acts to form ferritin-containing exosomes, which are exported from the cell, thereby preventing ferroptosis [54]. Meanwhile, the autophagic degradation of ferritin, a process known as ferritinophagy, is mediated by nuclear receptor co-activator 4 (NCOA4) and can enhance intracellular iron (Fe^2+^) levels, ultimately resulting in ferroptosis [55,56]. Furthermore, the Nrf2-regulated gene HO-1 catabolizes heme into three products: carbon monoxide, biliverdin, and free iron. By supplementing iron, HO-1 can effectively augment ferroptosis induced by erastin [52].

Mitochondrial proteins including cysteine desulfurase (NFS1), iron-sulfur cluster assembly enzyme (ISCU), CDGSH iron sulfur domain 1 (CISD1, also known as mitoNEET), and CISD2 (also known as nutrient-deprivation autophagy factor-1 (NAF-1)) are involved in the functional utilization of iron for iron-sulfur cluster biogenesis, acting to inhibit ferroptosis by increasing the biosynthesis of iron-sulfur clusters (Fe-S), thereby reducing intracellular iron levels [57,58,59,60] (Figure 2). An intracellular iron excess promotes subsequent lipid peroxidation by way of at least two mechanisms: (1) the iron-dependent Fenton reaction that produces reactive oxygen species (ROS); and (2), the activation of iron-containing enzymes such as lipoxygenases (ALOXs) [24,61,62]. In the process of ferroptosis, PUFAs are the most susceptible to lipid peroxidation, which can damage the membrane structure [63]. Acyl-CoA synthetase long chain family members (ACSLs) and lysophospholipid acyltransferase 3 (LPCAT3) promote the integration of polyunsaturated fatty acids (PUFAs) into phospholipids (PLs) to form polyunsaturated fatty acid-containing phospholipids (PUFA-PLs), which are sensitive to ROS-initiated oxidation mediated by ALOXs, leading to the formation of lipid peroxides (PUFA-PL-OOH), and ultimately ferroptosis [64,65,66,67].

Iron regulatory proteins, including aconitase 1 (ACO1) and iron-responsive element binding protein 2 (IREB2), play central roles in regulating cellular iron homeostasis at the posttranscriptional level. ACO1 is a Fe-S cluster protein that exists in two forms. When a cell is rich in iron, ACO1 exists in the form of cytoplasmic aconitase. When intracellular iron is lacking, ACO1 presents in the Fe-S cluster as a regulator of translation. Unlike ACO1, IREB2 is mainly regulated by F-box and leucine-rich repeat protein 5 (FBXL5)-mediated protein degradation. IREB2 undergoes degradation when iron is excessive and stabilizes when iron is lacking [68] (Figure 2). In the context of erastin-stressed cell death, IREB2 can relay the pro-ferroptosis effect by altering genes accounting for iron metabolism [69]. Mechanistically, ACO1/IREB2 acts to target the iron-responsive elements (IREs) which are conserved hairpin structures located in the untranslated regions (UTRs) of mRNAs. The main iron-regulating mRNAs can be regulated by ACO1/IREB2, including the genes involved in iron import (e.g., TFRC and SLC11A2), storage (e.g., FTH1 and FTL), and export (e.g., SLC40A1). Interestingly, the binding of ACO1/IREB2 to IREs located in the 5′ UTR and 3′ UTR has the opposite effect. The binding of ACO1/IREB2 to 5′ IRE leads to the inhibition of translation of mRNA; whereas the binding to 3′ IRE causes the promotion of translation of mRNA by inhibiting the degradation of mRNA. As shown in Figure 2, under the condition of iron deficiency, ACO1/IREB2 binds to the 3′ IREs of TFRC and SLC11A2 mRNAs, and the 5′ IREs of SLC40A1 and FTH1/FTL mRNAs. As a result, ACO1/IREB2 decreases the synthesis of TFRC and SLC11A2, while it increases the synthesis of SLC40A1 and FTH1/FTL mRNAs. On the other hand, an excess of cytosolic iron results in translational inhibition of TFRC and SLC11A2, and translational activation of SLC40A1 and FTH1/FTL [70]. Ferroptosis can be regulated by several transcription factors that control the transcription activity of genes involved in iron metabolism [71]. For instance, nuclear factor erythroid 2 like 2 (NFE2L2, also known as NRF2) plays a primary role in the anti-ferroptotic effect by up-regulating genes responsible for iron storage, such as FTH1/FTL [71,72] and SLC40A1 [73]. 

## 3. Antioxidant Mechanisms in Preventing Ferroptosis

**Figure 3:**
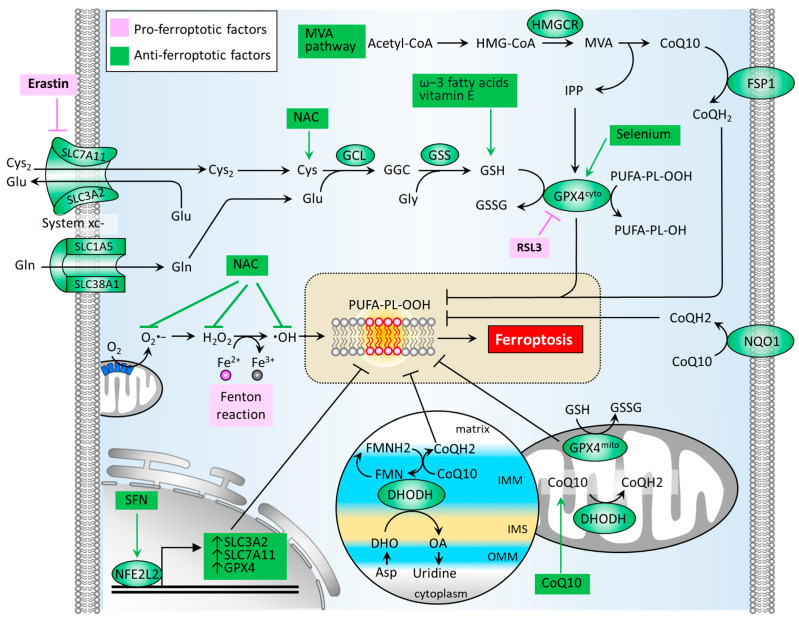
Molecular pathways of the antioxidant defense system that regulates ferroptosis. An antioxidant defense system is composed of GSH-dependent pathways (GSH/GPX4 and MVA) and GSH-independent pathways (FSP1/CoQ10 and DHODH/CoQ10). CoQ10, coenzyme Q10; CoQH2, reduced form of CoQ10; Cys, cysteine; Cys2, cystine; DHO, dihydroorotate; DHODH, dihydroorotate dehydrogenase; FMN, flavin mononucleotide; FMNH2, reduced form of FMN; FSP1, ferroptosis suppressor protein 1; GCL, glutamate-cysteine ligase; GPX4, glutathione peroxidase 4; GSH, glutathione; GSS, glutathione synthetase; GSSG, glutathione disulfide; HMGCR, HMG-CoA reductase; IMM, inner membrane of mitochondria; IMS, intermembrane space; IPP, isopentenyl-pyrophosphate; MVA, mevalonate; NAC, N-acetylcysteine; NFE2L2, nuclear factor, erythroid 2 like 2; NL-1, CISD1-targeting drug; NQO1, NAD(P)H quinone dehydrogenase 1; OA, orotate; OMM, outer membrane of mitochondria; ROS, reactive oxygen species; SFN, sulforaphane; SLC1A5, solute carrier family 1 member 5; SLC3A2, solute carrier family 3 member 2; SLC38A1, solute carrier family 38 member 1; SLC7A11, solute carrier family 7 member 11.

ROS or oxidative stress contributes significantly to the induction of Fenton reaction and the formation of lipid peroxides. Thus, the antioxidant defense system is a crucial factor in neutralizing ferroptosis development. Several classes of antioxidant pathways exist which can counteract ferroptosis, including the glutathione (GSH)-dependent phospholipid hydroperoxidase glutathione peroxidase 4 (GPX4) pathway in the cytosol (GPX4^cyto^) and mitochondria (GPX4^mito^). Additionally, the GSH-independent coenzyme Q10 (CoQ10, also known as ubiquinone) pathway is underpinned by ferroptosis suppressor protein 1 (FSP1, also known as apoptosis-inducing factor mitochondrial 2 (AIF-M2)) at the plasma membrane (FSP1-CoQ10 axis) and dihydroorotate dehydrogenase (DHODH) in the inner membrane of mitochondria (DHODH-CoQ10 axis) (Figure 3) [74,75]. 

The synthesis of GSH primarily relies on the import of cystine (Cys_2_). System xc^−^ is a cystine/glutamate antiporter which is widely distributed in phospholipid bilayers and acts to import Cys_2_ into cells with a 1:1 counter-transport of glutamate [9,22], and maintains homeostasis of the cellular antioxidant system. System xc^−^ is a heterodimer composed of two subunits: solute carrier family 7 member 11 (SLC7A11) and solute carrier family 3 member 2 (SLC3A2). The Cys_2_ within cells can be oxidized to cysteine (Cys) in a reaction catalyzed by glutamate-cysteine ligase (GCL) and glutathione synthetase (GSS), which is required for the synthesis of GSH [24]. The GSH functions to reduce ROS and reactive nitrogen under the activity of glutathione peroxidases. Among the GPX family, GPX4 plays a critical role in regulating the manifestation of ferroptosis. The biosynthesis of GPX4 requires the micronutrient selenium [76]. GPX4 can convert GSH into oxidized glutathione (GSSG) and reduce cytotoxic lipid peroxides (L-OOH) to the corresponding alcohols (L-OH), thus hindering the formation of lipid peroxides (Figure 3). Several GPX4 inhibitors have been reported to stimulate cell ferroptosis, including RSL3, ML162, ML 210, FIN56, FINO_2_ [75]. By generating anti-ferroptotic biomolecules including isopentenyl-pyrophosphate (IPP) and CoQ10, the mevalonate (MVA) pathway counteracts ferroptosis. The synthetic processes of the two molecules require the rate-limiting enzyme, HMG-CoA reductase (HMGCR), which is also an inhibitory target of statins (a class of cholesterol-lowering drugs) [77]. IPP acts to stabilize selenocysteine tRNA, which is required for the synthesis of GPX4 [78]. As for the GPX4-independent CoQ10 pathway, Bersuker et al. first reported that FSP-1, a flavoprotein formerly known as AIF-M2 (apoptosis-inducing factor mitochondrial 2), exhibits a protective effect against the generation of ferroptosis, as induced by GPX4 deletion [79]. At the plasma membrane, FSP-1 acts as an oxidoreductase that reduces CoQ10 to generate CoQH2 (also known as ubiquinol) which can repair lipid peroxides [80]. Aside from FSP-1, NAD(P)H quinone dehydrogenase 1 (NQO1) in the plasma membrane acts to reduce CoQ10 to generate CoQH2 [81], offering an alternative pathway to prevent lipid peroxidation. A more recent report by Mao et al. reveals that the mitochondrial enzyme DHODH acts to coordinate with GPX4^mito^, inhibiting ferroptosis by detoxifying lipid peroxides accumulated in mitochondria [74]. DHODH, an iron-containing flavin-dependent enzyme, is involved in the de novo synthesis of pyrimidines within mitochondria [82]. As reported, DHODH generates CoQH2 by reducing CoQ10 through a uridine-synthesizing redox reaction which catalyzes dihydroorotate to orotate [74]. Of note is that DHODH inhibitors have been applied in the treatment of autoimmune diseases, such as multiple sclerosis and rheumatoid arthritis [75]. Although published reports involving the role of DHODH-mediated CoQH2 generation in PD remain limited, the use of CoQ10 supplementation in patients with PD has demonstrated safety and patient tolerance, achieving phase 2/3 trials. With regard to transcriptional control, NFE2L2 acts to up-regulate GPX4, SLC7A11, and SLC3A2, thereby boosting the antioxidant defense system [71]. 

## 4. Targeting Ferroptosis in the Treatment of PD

A growing body of evidence supports the therapeutic role of targeting iron accumulation in the treatment of PD. Deferiprone (DFP) presents iron-scavenging activity [83], especially for iron accumulation in mitochondria, the organelle considerably affected by iron-dependent oxidative damage in the context of neurodegeneration [84,85,86]. Devos et al. demonstrated that DFP supplementation (30 mg/kg/day) to PD patients exhibited efficacy and safety in a pilot double-blind, placebo-controlled randomized clinical trial (RCT) [29]. A phase 2 RCT conducted by Martin-Bastida et al. revealed that DFO exerted iron-lowering effects in some areas of the brain, leading to a nonsignificant improvement of motor functions according to the Unified Parkinson’s Disease Rating Scale (UPDRS) [30]. A widely used antiparasitic agent, clioquinol (iodochlorhydroxyquin), has been reported to present redox-silencing of reactive Fe^2+^ and neuroprotective effects [87,88]. Shi et al. reported that clioquinol treatment improved motor and nonmotor deficits in an MPTP () intoxication monkey model [31]. Clioquinol treatment resulted in decreased iron accumulation and lipid peroxidation in the substantia nigra of the animals. Mitochondrial protein CISD1 is implicated in the pathophysiology of PD. Geldenhuys et al. demonstrated that CISD1-knockout mice showed increased ROS and cell loss of the dopaminergic neurons, resulting in impaired motor performance [89]. In respect to CISD1-targeting drugs, NL-1 is the first drug designed by removal of the tail of pioglitazone to eliminate PPAR-γ activity, while retaining CISD1 binding affinity [90]. Geldenhuys et al. demonstrated that NL-1 provided neuroprotection against the PD toxin rotenone [32]. Zuo et al. recently reported that ferritinophagy plays a critical role in inducing neurotoxicity [34]. The PD-inducing toxin, paraquat, causes iron accumulation in cytoplasm and mitochondria and increases arachidonate 12-Lipoxygenase (ALOX12) via NCOA4-involved ferritinophagy activity. The ferroptosis inhibitor Ferrostatin-1 (Fer-1) acts to reduce ALOX12 levels and reverses paraquat-induced ferroptosis [34]. Southon et al. revealed that Liproxstatin-1 (Lip-1) acts to prevent neuronal cell ferroptosis induced by RSL3/erastin [35]. Fernandez-Mendivil et al. reported that lipopolysaccharide (LPS) induced neuroinflammation and ferroptosis in the mouse brain, concurrent with increased HO-1 levels [33]. The inflammatory molecular phenotype was prevented both in mice with microglial HO-1 knockout, and in mice treated with HO-1 inhibitor ZnPP (zinc protoporphyrin) [33]. 

Eliminating lipid peroxides resulting from iron accumulation and ROS stress is an emerging therapeutic strategy. Carnosine, an endogenous histidyl dipeptide (β-alanine-l-histidine), acts to conjugate lipid peroxides to provide protective effects against oxidation damage [91]. In a 6-hydroxydopamine (6-OHDA)-insulted PD cellular model, carnosine was shown to reduce neuronal cell death and ROS production [92]. Brown et al. demonstrated that intranasal carnosine treatment reduced α-syn accumulation in the substantia nigra and motor function in a Thy1-α-syn PD mice model [93]. Carnosine has been reported to increase the effectiveness of PD patients’ primary therapy, decrease the UPDRS score, and restore superoxide dismutase (SOD) levels in a phase 1 clinical trial [36]. Cu^II^(atsm) is a bis(thiosemicarbazone)copper^II^ compound which has been found to reduce iron overload-induced lipid peroxidation and prevent cell ferroptosis [35]. Hung et al. found that Cu^II^(atsm) rescued dopaminergic cell loss in both MPTP (1-methyl-4-phenyl-1,2,3,6-tetrahydropyridine) PD mouse model and a human A53T (hA53T) mutant α-syn overexpression PD mouse model [37]. Importantly, Cu^II^(atsm) recently presented promising preliminary results in both a phase 1 trial of patients with amyotrophic lateral sclerosis (NCT02870634) and a phase 1 trial of patients with PD (NCT03204929). Neurotoxin 6-OHDA increases the levels of ACO1 in 6-OHDA lesioned rats, while inhibition of ACO1 leads to neuroprotection [94]. 

In terms of approaches to target antioxidant defense, an increasing number of pre-clinical settings and clinical trials demonstrate their safety and efficacy. The clinical benefit and safety of CoQ10 supplementation has been reported in several phase 2 clinical trials [38,39], while some clinical trials exhibited no significant benefit [95]. Mitochondria-targeting CoQ10 (MitoQ) presented no clinical benefit in slowing PD progression in a phase 2 clinical trial [96]. Ellwanger et al. reported that paraquat-caused locomotor impairment in rats can be restored by selenium feeding [40]. Additionally, Sepidarkish et al. reported in a systemic review [97] that omega-3 fatty acid with vitamin E co-supplementation enhances total antioxidant capacity and decreases oxidative damage. In a randomized, double-blind placebo-controlled clinical trial, the oral adjunction of omega-3 fatty acids and vitamin E for three months improved GSH level and motor scale UPDRS [41]. Furthermore, a phase 1/2a clinical study conducted by Mischley et al. demonstrated that intranasal GSH supplement was safe and well-tolerated over a three-month intervention period [42]. However, phase 2b of the study showed no significant difference from the placebo group [43]. The fact that one participant developed cardiomyopathy [43] may delay future studies. In addition, NAC (N-acetylcysteine) is a synthetic derivative of the endogenous amino acid L-cysteine, a GSH precursor. NAC has been prescribed for replenishment of hepatic GSH after acetaminophen overdose [98]. The mechanisms accounting for NAC’s antioxidant activity may lie in supplementing GSH synthesis, scavenging ROS, and detoxification of semiquinones, hypochlorous acid (HOCl), nitroxyl (HNO), and heavy metals [99]. Two phase 1 trials reported that NAC administration boosted GSH redox ratios, brain GSH, and cerebrospinal fluid (CSF) NAC concentrations [44,45]. Meanwhile, Sulforaphane (SFN) is an isothiocyanate found in cruciferous vegetables, identified as an NFE2L2 inducer. SFN has been successfully used in clinical trials for the treatment of patients with type II diabetes mellitus [100,101]. In in vivo PD mouse models, SFN acted toward preventing neurotoxin-induced dopaminergic neuron loss [46,47,48]. 

## 5. Role of Ferroptosis in Microglia/Macrophage M1/M2 Polarization

**Figure 4:**
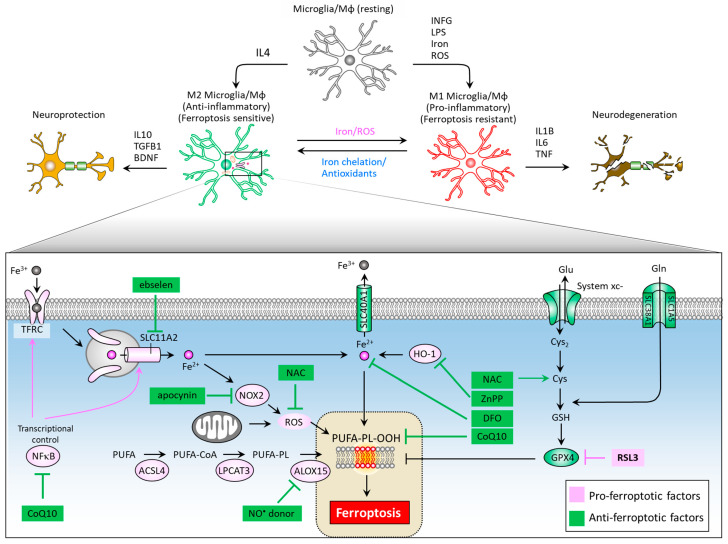
Ferroptosis-associated molecules at the crossroad of microglia/macrophage M1/M2 polarization and neuroinflammation. While M1 microglia feature ferroptotic resistance, M2 microglia are vulnerable to ferroptosis. Therapeutic targets to reduce iron accumulation, scavenge ROS, and impede the formation of lipid peroxides could provide new hope in the treatment of PD and neurodegenerative disorders. ACSL4, acyl-CoA synthetase long chain family member 4; ALOX15, arachidonate 15-lipoxygenases; BDNF, brain derived neurotrophic factor; CoQ10, coenzyme Q10; Cys, cysteine; Cys2, cystine; DFO, desferoxamine; DHO, dihydroorotate; DHODH, dihydroorotate dehydrogenase; Gln, glutamine; Glu, glutamate; GPX4, glutathione peroxidase 4; GSH, glutathione; HO-1, heme oxygenase-1; IFNG, interferon gamma; IL1B, interleukin-1 beta; IL4, interleukin-4; IL10, interleukin-10; LPS, lipopolysaccharide; Mφ, macrophage; NAC, N-acetylcysteine; NFκB, nuclear factor kappa B; NOX2, NADPH oxidase 2; PUFA-PL-OOH, lipid peroxides generated from polyunsaturated fatty acid-containing phospholipids; ROS, reactive oxygen species; SLC1A5, solute carrier family 1 member 5; SLC3A2, solute carrier family 3 member 2; SLC11A2, solute carrier family 11 member 2; SLC40A1, solute carrier family 40 member 1; SLC7A11, solute carrier family 7 member 11; TFRC, transferrin receptor; TGFB1, transforming growth factor beta 1.

Neuroinflammation mediated primarily by resident brain microglia has gained attention from researchers as both an important mediator of the dopaminergic neuron loss in PD and an attractive drug target for neurodegenerative disease therapy [102,103,104,105]. Microglia are the cells responsible for mediating the innate immune response in the brain through antigen-presenting and effector functions such as phagocytosis [106]. When microglia are activated, transformation and proliferative events occur to form reactive microglia [107], which are distinguished by two distinct phenotypes: the M1 phenotype (pro-inflammatory), and the M2 phenotype (anti-inflammatory). Rodriguez-Perez et al. showed that overexpression of α-syn induces a significant increase in M1 marker expression and decreased M2, as well as a marked dopaminergic neuron loss and impaired motor function in mice. They also demonstrated that deactivated microglia through inhibition of angiotensin type 1 receptors leads to decreased M1 and increased M2 marker expressions, as well as restored dopaminergic neurons and ameliorated motor function [108]. Interestingly, microglial activation can affect intercellular transfer of α-Syn. George et al. demonstrated that microglia play a role in α-syn cell-to-cell transfer, and that anti-inflammatory microglia may enhance clearance of α-syn from extracellular space [109]. The M1 phenotype can be induced by LPS, IFNγ, iron and ROS exposure, and is characterized by increased iNOS expression and the secretion of inflammatory cytokines such as IL6, IL1β, and TNF, leading to neurodegeneration (Figure 4). On the other hand, M2 phenotype differentiation can be achieved by IL4 treatment, and is characterized by increased arginase-1 levels and secretion of the brain-derived neurotrophic factor (BDNF), and anti-inflammatory cytokines such as IL10 and TGFB, leading to neuroprotection. 

Intracellular iron levels modulate differentiation towards one or the other phenotype [110]. An iron overload triggers M1 polarization via an ROS-mediated mechanism [111], increasing TNF and IL1B secretion [112], and causes M2 macrophages to switch their phenotype to M1 [113]. Iron chelator deferoxamine reduces ROS levels and TNF and IL1B secretion by microglia [114] and also promotes microglial M2 polarization neurodegeneration in animals [115]. Kapralov et al. revealed that M1 microglia are resistant to ferroptotic stress, while M2 are more sensitive [116]. M2 microglia express remarkably lower levels of inducible nitric oxide synthase (iNOS) than M1 microglia. Specifically, the ferroptotic resistance of M1 microglia requires iNOS/NO^•^, while NO^•^ empowers the resistance of M2 microglia to ferroptosis. The activation of nuclear factor kappa B (NFκB), a master transcription factor of neuroinflammation, serves to enhance the expression of SLC11A2, contributing to iron accumulation in dopaminergic neurons [117]. Of note, ebselen, a SLC11A2 inhibitor was noted to reduce iron accumulation in the substantia nigra of a neuroinflammation mouse model and improve motor performance [118]. Iron overload can elicit microglial activation and promote NADPH oxidase 2 (NOX2)-dependent ROS generation, further contributing to iron-mediated ferroptosis in midbrain-derived primary cultures [119]. NOX2 activation is implicated in paraquat-mediated microglial activation by iron [120]. The NOX inhibitor apocynin acts to increase the expression of SLC40A1, inhibit iron accumulation and lipid peroxidation, alleviate neuroinflammation, and recue dopaminergic neuron loss [121]. In an LPS/iron-induced neuroinflammation cell model, NAC as an ROS scavenger effectively suppressed the expression of proinflammatory cytokine [110], indicating NAC’s role in modulating iron-mediated neuroinflammation. In addition, inhibition of HO-1 activity by ZnPP can abolish neuroinflammation in the mouse brain [33]. Yan et al. reported that a CoQ10 analog, idebenone, exhibits a suppressive effect on neuroinflammation microglial phenotype in an MPTP-stressed PD mouse model by inhibiting Mitogen-Activated Protein Kinase 1/3 (MAPK1/3) and the NFκB signaling pathway [122]. Figure 4 illustrates a summary diagram of ferroptosis pathways and corresponding therapeutic targeting in M1/M2 microglia polarization and neuroinflammation.

## 6. Cerebrospinal Fluid (CSF) and Blood Biomarkers

Given the importance of ferroptosis as an emerging pathway in the pathogenesis of PD, and in light of new therapies, the understanding of ferroptosis-related biomarkers can be of translational value. Table 3 shows a nonexhaustive list of ferroptosis-related biomarkers of important in the context of PD. Isobe et al. reported that the percentage of oxidized/total CoQ10 in the CSF was significantly higher in the PD group compared to the normal control group [123]. Maarouf et al. found the postpartum CSF of PD had lower levels of glutathione S-transferase pi 1 (GSTP1) compared to normal control subjects [124]. Lewitt et al. demonstrated a decrease in CSF concentrations of GSH compared to normal control [125]. Yu et al. reported that higher **∙**OH levels of CSF in PD patients than controls [126]. Boll et al. found the higher levels of lipid peroxidation products in the CSF PD patients than normal controls [127]. A systemic review conducted by Wei et al. revealed higher blood concentrations of malondialdehyde (MDA, a final product of lipid peroxidation) and ferritin, and lower blood concentrations of GSH in PD than in healthy control subjects [128]. Charisis et al. showed that a 1 μmol/L increase in plasma GSH was associated with 0.4% less increase in prodromal PD probability for 1 year of follow-up [129]. 

## 7. Conclusions

Advanced investigations have provided further insight into our understanding of ferroptosis, which involves the integration of highly organized systems that regulate iron metabolism, lipid peroxidation, and antioxidant defense. More importantly, clinical trials that apply ferroptosis-counteracting agents to patients with PD are ongoing. As such, it is inevitable that continued research in this field will further elucidate the physiological and pathological roles of ferroptosis, leading to the development of translational strategies for the treatment of neurodegenerative diseases, including PD. However, to unequivocally monitor the therapeutic efficacy of future ferroptosis-targeting drug candidates, new ferroptosis-specific pharmacodynamic biomarkers are urgently required and await discovery.

## Figures and Tables

**Table 2:** Updated therapeutic approaches targeting ferroptosis pathways in neuroinflammation and PD.

Mechanism of Action	Agent	Phase of Clinical Development	Reference
↓Iron (chelating)	DFP	Phase 2	NCT00943748 [29]
↓Iron (chelating)	DFP	Phase 2	NCT01539837 [30]
↓Iron (chelating)	DFP	Phase 2	NCT02728843
↓Iron (chelating)	DFP	Phase 2	NCT02655315
↓Iron (chelating)	Clioquinol	in vivo	[31]
↓Iron (targeting CISD1)	NL-1	in vitro	[32]
↓HO-1	ZnPP	in vivo	[33]
↓Lipid peroxides	Fer-1	in vitro	[34]
↓Lipid peroxides	Lip-1	in vitro	[35]
↓Lipid peroxides	Carnosine	Phase 1	[36]
↓Lipid peroxides	Cu^II^(atsm)	in vitro; in vivo	[35,37]
↓Lipid peroxides	Cu^II^(atsm)	Phase 1	NCT03204929
↑Antioxidant defense	Ubiquinone (CoQ10)	Phase 2	[38,39]
↑Antioxidant defense	Selenium	in vivo	[40]
↑Antioxidant defense	Omega 3 fatty acids, vitamin E	Phase 2	[41]
↑Antioxidant defense	GSH	Phase 1/2a, 2b	[42,43]
↑Antioxidant defense	NAC	Phase 1	[44,45]
↑Antioxidant defense (↑NFE2L2)	SFN	in vivo	[46,47,48]

↑, promote; ↓, inhibit; CoQ10, coenzyme Q10; Cu^II^ (atsm), diacetyl-bis(4-methyl-3-thiosemicarbazonato) copper^II^; DFP, deferiprone; Fer-1, Ferrostatin-1; GSH, glutathione; HO-1, heme oxygenase-1; Lip-1, Liproxstatin-1, NAC, N-acetylcysteine; NFE2L2, nuclear factor erythroid 2 like 2; PUFA-PL-OOH, lipid peroxides generated from polyunsaturated fatty acid-containing phospholipids; SFN, sulforaphane; ZnPP, zinc protoporphyrin.

**Table 3:** Ferroptosis-related biomarkers of PD.

Source	Biomarker	Expression in PD	References
CSF	Oxidized Q10	↑	[123]
CSF	GSTP1	↓	[124]
CSF	GSH	↓	[125]
CSF	**∙**OH	↑	[126]
CSF	lipid peroxidation	↑	[127]
Blood	lipid peroxidation (MDA)	↑	[128]
Blood	ferritin	↑	[128]
Blood	GSH	↓	[128]
Blood	GSH	↓	[129]

CSF, cerebrospinal fluid; GSH, glutathione; GSTP1, glutathione S-transferase pi 1; MDA, malondialdehyde; **∙**OH, hydroxyl radical.

## Data Availability

No new data were created or analyzed in this study. Data sharing is not applicable to this article.

## Abbreviations

6-OHDA	6-hydroxydopamine
α-syn	α-synuclein
ACO1	aconitase 1
ACSL4	acyl-CoA synthetase long chain family member 4
AIF-M2	apoptosis-inducing factor mitochondrial 2
AIM2	absent in melanoma like receptor 2
ALOXs	arachidonate lipoxygenases
APAF1	apoptotic peptidase activating factor 1
ASC	apoptosis-associated speck-like protein containing a CARD
BAD	BCL2 associated agonist of cell death
BAK	Bcl-2 homologous antagonist/killer
BAX	Bcl-2-associated X
BBC3	BCL2 binding component 3
BCL2	B-cell lymphoma 2
BCL2L11	BCL2 Like 11
BID	BH3 interacting domain death agonist
BDNF	brain-derived neurotrophic factor
CISD1	CDGSH iron sulfur domain 1
CISD2	CDGSH iron sulfur domain 2
Cys	cysteine
Cys2	cystine
DFP	deferiprone
DFX	deferoxamine
DHO	dihydroorotate
DHODH	dihydroorotate dehydrogenase
FADD	Fas-associated protein with death domain
FBXL5	F-box and leucine-rich repeat protein 5
FDA	Food and Drug Administration
Fer-1	Ferrostatin-1
FSP-1	ferroptosis suppressor protein 1
FTH1	ferritin heavy chain 1
FTL	ferritin light chain
GCL	glutamate-cysteine ligase
GPX4	glutathione peroxidase 4
GSDMD	gasdemin D
CSF	cerebrospinal fluid
GSH	glutathione
GSS	glutathione synthetase
HMGCR	HMG-CoA reductase
HNO	nitroxyl
HO-1	heme oxygenase-1
HOCl	hypochlorous acid
ICD	immunogenic cell death
IFNG	interferon gamma
iNOS	inducible nitric oxide synthase
IL1B	interleukin-1 beta
IL4	interleukin-4
IL10	interleukin-10
IL18	interleukin-18
IMM	inner membrane of mitochondria
IMS	intermembrane space
IPP	isopentenyl-pyrophosphate
IREB2	iron-responsive element binding protein 2
IREs	iron-responsive elements
ISCU	iron-sulfur cluster assembly enzyme
Lip-1	Liproxstatin-1
LPCAT3	lysophosphatidylcholine acyltransferase 3
LPS	lipopolysaccharide
LTF	lactotransferrin
Mf	macrophage
MAPK1	mitogen-activated protein kinase 1
MAPK3	mitogen-activated protein kinase 3
MLKL	mixed lineage kinase domain-like pseudokinase
MOMP	mitochondrial outer membrane permeabilization
MVA	mevalonate
NAC	N-acetylcysteine
NCOA4	nuclear receptor co-activator 4
Nec-1	necrostatin-1
NFE2L2	nuclear factor erythroid 2 like 2
NFS1	cysteine desulfurase
NLRP3	nod-like receptor 3
NOX2	NADPH oxidase 2
NQO1	NAD(P)H quinone dehydrogenase 1
OA	orotate
OMM	outer membrane of mitochondria
PD	Parkinson’s disease
PMAIP1	phorbol-12-myristate-13-acetate-induced protein 1
PROM2	prominin2
PUFAs	polyunsaturated fatty acids
PUFA-PL-OOH	lipid peroxides generated from polyunsaturated fatty acid-containing phospholipids
RCD	regulated cell death
RCT	randomized clinical trial
RIP1	receptor-interacting protein kinase 1
RIP3	receptor-interacting protein kinase 3
ROS	reactive oxygen species
SFN	sulforaphane
SLC1A5	solute carrier family 1 member 5
SLC3A2	solute carrier family 3 member 2
SLC38A1	solute carrier family 38 member 1
SLC7A11	solute carrier family 7 member 11
SLC11A2	solute carrier family 11 member 2
SLC40A1	solute carrier family 40 member 1
SCL46A1	solute carrier family 46 member 1
SCL48A1	solute carrier family 48 member 1
SOD	superoxide dismutase
TF	transferrin; TFRC, transferrin receptor
tBID	truncated BH3 interacting domain death agonist
TCD	tolerogenic cell death
TF	transferrin
TFRC	transferrin receptor
TGFB1	transforming growth factor beta 1
TNF	tumor necrosis factor
TNFR	tumor necrosis factor receptor
TRAIL	tumor necrosis factor-related apoptosis-inducing ligand
TRAILR1	tumor necrosis factor-related apoptosis-inducing ligand receptor 1
ZnPP	zinc protoporphyrin
